# Prosthesis alignment affects axial rotation motion after total knee replacement: a prospective *in vivo* study combining computed tomography and fluoroscopic evaluations

**DOI:** 10.1186/1471-2474-13-206

**Published:** 2012-10-23

**Authors:** Melinda K Harman, Scott A Banks, Stephan Kirschner, Jörg Lützner

**Affiliations:** 1 Department of Bioengineering, Clemson University, 301 Rhodes Engineering Research Center, SC, Clemson, 29634-0905, USA; 2 Medical Technology Laboratory, Rizzoli Orthopaedic Institute, Via di Barbiano 1/10, Bologna, 40136, Italy; 3 Department of Mechanical & Aerospace Engineering, University of Florida, P.O. Box 116250, Gainesville, FL, 32611-6250, USA; 4 Orthopaedic Department, University Hospital Carl Gustav Carus Dresden, Fetscherstrasse 74, Dresden, 01307, Germany

**Keywords:** Total knee replacement, Mobile-bearing prosthesis, Implant alignment, Surgical alignment, Knee kinematics, Axial rotation, Knee biomechanics, Knee arthroplasty

## Abstract

**Background:**

Clinical consequences of alignment errors in total knee replacement (TKR) have led to the rigorous evaluation of surgical alignment techniques. Rotational alignment in the transverse plane has proven particularly problematic, with errors due to component malalignment relative to bone anatomic landmarks and an overall mismatch between the femoral and tibial components’ relative positions. Ranges of nominal rotational alignment are not well defined, especially for the tibial component and for relative rotational mismatch, and some studies advocate the use of mobile-bearing TKR to accommodate the resulting small rotation errors. However, the relationships between prosthesis rotational alignment and mobile-bearing polyethylene insert motion are poorly understood. This prospective, *in vivo* study evaluates whether component malalignment and mismatch affect axial rotation motions during passive knee flexion after TKR.

**Methods:**

Eighty patients were implanted with mobile-bearing TKR. Rotational alignment of the femoral and tibial components was measured from postoperative CT scans. All TKR were categorized into nominal or outlier groups based on defined norms for surgical rotational alignment relative to bone anatomic landmarks and relative rotational mismatch between the femoral and tibial components. Axial rotation motion of the femoral, tibial and polyethylene bearing components was measured from fluoroscopic images acquired during passive knee flexion.

**Results:**

Axial rotation motion was generally accomplished in two phases, dominated by polyethylene bearing rotation on the tibial component in early to mid-flexion and then femoral component rotation on the polyethylene articular surface in later flexion. Opposite rotations of the femur-bearing and bearing-baseplate articulations were evident at flexion greater than 80°. Knees with outlier alignment had lower magnitudes of axial rotation and distinct transitions from external to internal rotation during mid-flexion. Knees with femoral-tibial rotational mismatch had significantly lower total axial rotation compared to knees with nominal alignment.

**Conclusions:**

Maintaining relative rotational mismatch within ±5° during TKR provided for controlled knee axial rotation during flexion. TKR with rotational alignment outside of defined surgical norms, with either positive or negative mismatch, experienced measurable kinematic differences and presented different patterns of axial rotation motions during passive knee flexion compared to TKR with nominal mismatch. These findings support previous studies linking prosthesis rotational alignment with inferior clinical and functional outcomes.

**Trial Registration:**

Clinical Trials NCT01022099

## Background

Attaining proper prosthesis alignment during total knee replacement (TKR) is essential for stable TKR function and successful clinical outcomes [1-5]. The associated technical challenges have led to the rigorous evaluation of surgical alignment techniques for identifying anatomic landmarks and defining the joint axes during TKR surgery [6-13]. However, deviation from optimal alignment persists in some cases, especially in the transverse plane (rotational alignment) [14-17]. Furthermore, optimal rotational alignment of the femoral and tibial components relative to fixed anatomic landmarks can still produce complications due to an overall mismatch in rotational alignment of the femoral component relative to the tibial component [14,16,18-20].

TKR designs with mobile polyethylene bearings are advocated for their professed ability to self-align and accommodate small rotational alignment errors [19,21-24]. Such errors can include rotational malalignment with respect to bone anatomic landmarks, as well as mismatch between the relative positions of the femoral and tibial components. However, for many mobile-bearing TKR, understanding the relationships between prosthesis rotational alignment, knee axial rotation motion, and polyethylene bearing motion is difficult [24-28]. Consequently, it remains largely unknown whether knee axial rotation is accomplished through femoral component motion on the bearing articular surface or through bearing motion on the tibial baseplate.

This study addresses the following specific research question. Does component malalignment affect knee axial rotation motion and bearing motion in mobile-bearing TKR? The objective was to assess TKR rotational alignment, knee axial rotation motion and polyethylene bearing motion that occur *in vivo* during passive flexion in subjects with mobile-bearing TKR. It was hypothesized that TKR with rotational alignment within defined surgical norms would present different knee axial rotation motion and bearing motion compared to TKR with rotational alignment outside surgical norms.

## Methods

The study protocol was approved by the local Ethics Commission at the clinical site and the National Board for Radiation Safety. Subjects were recruited from the hospital of the surgeon authors as part of a prospective, randomized study of TKR alignment described in detail elsewhere [15]. A total of 80 subjects met all surgical inclusion criteria and provided written informed consent to participate (Table 1). All subjects were operated by two arthroplasty fellowship trained surgeons (SK, JL) and implanted with a cruciate-retaining mobile-bearing TKR with a rotating platform polyethylene bearing (Scorpio^TM^ PCS, Stryker Orthopaedics, Mahwah, NJ, USA). The bearing thickness was 10 mm in 58 TKR and 12 mm in 22 TKR. No patellar resurfacing was performed and the tibial and femoral components were fixed with cement in all cases. The surgical technique for all TKR referenced the knee joints’ anatomical and mechanical axes, as well as anatomic landmarks detected intraoperatively by the surgeon [15]. The ability to surgically achieve the target alignments was assessed in a previous study by the surgeon authors [15], reporting that median deviation for femoral component alignment was ≤ 1.8° in all planes, and median deviation from tibial component alignment was ≤ 1.3° in the frontal and sagittal planes and ≤ 6.0° in the transverse plane. 

**Table 1:** Patient demographics and preoperative clinical data for entire randomized subject population and cohort included in the kinematic analysis (medians and range for continuous data, absolute and relative frequencies for categorical data)

	**All subjects**	**Kinematic cohort**
**n**	80	67
**Age (years)**	69 (47 – 87)	69 (47 – 84)
**Sex (% female)**	51/80, 63.8%	41/67, 65.7%
**Weight (kg)**	84 (60 – 146)	85 (62 – 146)
**Body Mass Index (kg/m**^**2**^**)**	29.7 (22.0 – 47.7)	30.0 (22.0 – 47.7)

**Figure 1:**
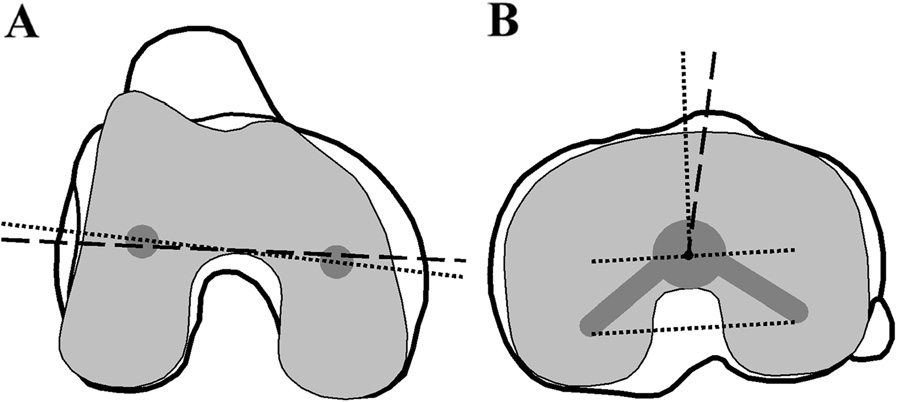
**The Anatomic Landmarks groups were distinguished by component alignment relative to anatomic axes. **(**A**) Femoral component alignment was measured as the angle between an axis defined by the femoral fixation pegs (dotted line) and the surgical epicondylar axis (dashed line), with nominal alignment within ± 3° or outlier alignment exceeding 3°. (**B**) Tibial component alignment was measured as the angle between an axis perpendicular to the posterior border of the tibial fixation keel projected to the tibial post center (dotted line) and an axis connecting the tibial post center and the medial third of the tibial tuberosity (dashed line), with nominal alignment within ± 10° or outlier alignment exceeding 10°.

Three quantitative descriptors of the components’ rotational alignment were measured (ID.PACS 3.6, Image Devices, Idstein, Germany) from computed tomography (CT) images of the knee in extension acquired for each patient 5 to 7 days after surgery. Rotational alignment with respect to anatomic landmarks was measured for the femoral component relative to the surgical transepicondylar axis and for the tibial component relative to the medial third of the tibial tuberosity (Figure 1), as previously described [15]. Relative rotational mismatch between the femoral and tibial components was measured by superimposing the CT images and measuring the angular divergence of the femoral component relative to the tibial component. This procedure of using commercially available software to measure prosthesis alignment from CT images was selected because it has very good intraobserver accuracy (intraclass correlation coefficient > 0.8) and coefficient of variation of 11% to 17% [29,30]. All CT measurements were completed by the same skilled observer (JL) who was blinded to kinematic measurements. 

Based on these CT measurements, all TKR were categorized according to nominal tolerances for surgical rotational alignment using criteria based on bone anatomy (Anatomic Landmarks group) or relative rotational alignment between the femoral and tibial components (Rotational Mismatch group). The tolerances were established using both surgical norms and a clinical perspective, since acceptable tolerance for tibial rotational alignment and rotational mismatch between the components are not well-defined [10,15,16,18,20,31-34]. In the Anatomic Landmarks group, TKR were categorized as having “nominal” rotational alignment with respect to anatomic landmarks if the alignment was within ±3° for the femoral components and ±10° for the tibial components. TKR exceeding these limits were categorized as “outliers”. In the Rotational Mismatch group, TKR were categorized as having “nominal” rotational mismatch if the relative femoral-tibial rotational mismatch was within ±5°. TKR exceeding these limits were categorized as “outliers”.

**Figure 2:**
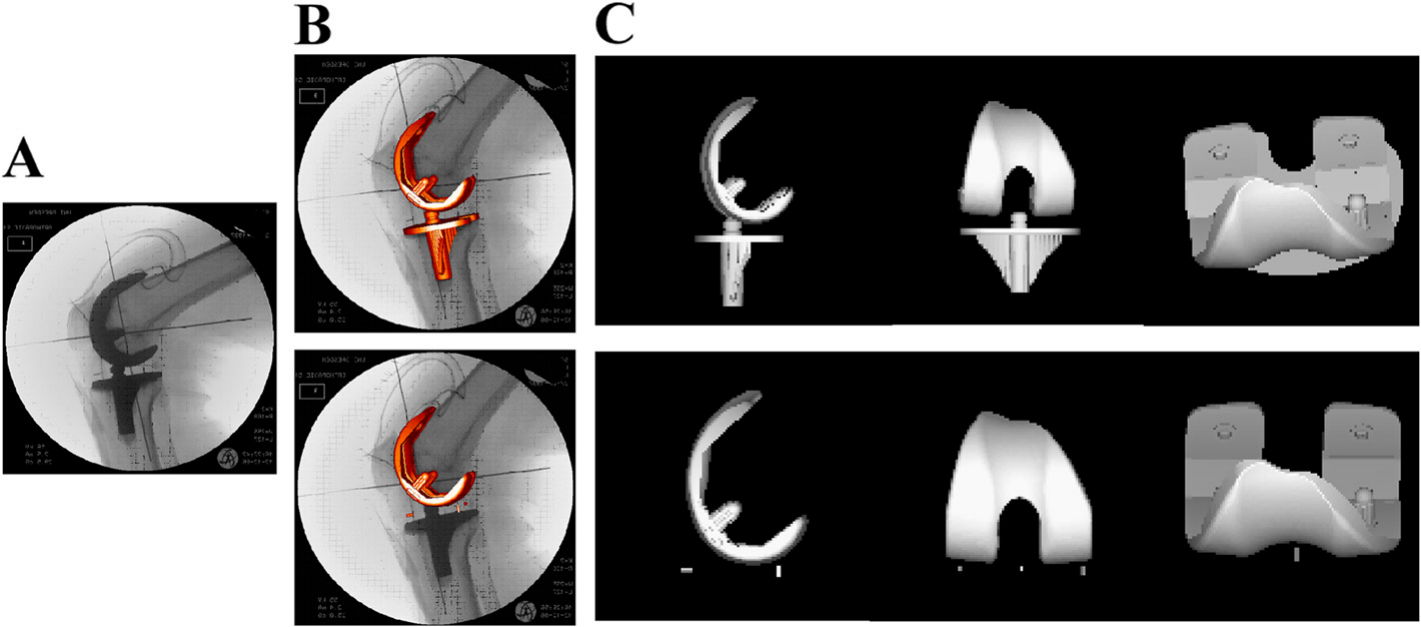
**Accurate measurement of three-dimensional TKR kinematics using fluoroscopy. **Model-based shape matching techniques were applied to (**A**) the acquired two-dimensional fluoroscopy images by (**B**) superimposing surface models of the components and embedded radiopaque markers and iteratively adjusting their three-dimensional pose to match the TKR silhouette. (**C**) Joint angles, including flexion, valgus, and axial rotation, were determined from the relative orientation between the femoral component and metal tibial baseplate and between the femoral component and polyethylene tibial bearing in each image.

TKR axial rotation motion during knee flexion was measured from fluoroscopic images (4 to 8 images per knee) acquired immediately after operative wound closure with the surgeon applying passive range of motion from full extension to approximately 120°. The three-dimensional position and orientation of femoral, tibial and polyethylene bearing components were determined using previously published model-based shape matching techniques (Figure 2) [25,35]. Three radiopaque markers embedded in each polyethylene bearing provided geometrically defined point clusters suitable for tracking bearing motion, as demonstrated in other studies [25,36-39]. Briefly, the measurement technique involved acquisition of two-dimensional fluoroscopy images, image calibration based on known dimensions of the imaging geometry (principal distance, beam center location), and projection of surface models of the prosthesis components and embedded radiopaque markers onto the fluoroscopic images with iterative adjustment of their three-dimensional pose to match the TKR silhouette. Joint angles, including flexion, valgus, and axial rotation, were determined from the relative orientation between the femoral component and metal tibial baseplate and between the femoral component and polyethylene tibial bearing in each image. Using images generated from sample femoral and tibial components mounted to a synthetic knee model and secured in a known orientation, error due to image distortion and matching was 0.3° for rotations and 1.0 mm for translations in the image plane. All kinematic assessments were completed by the same observer (MKH) who was blinded to the CT rotational alignment measurements. 

These measurements from fluoroscopic images determined the relative axial rotation motion for all three prosthesis components. Total knee axial rotation was defined as relative internal-external motion between the femoral component and metal tibial baseplate in the transverse plane. Articular axial rotation was defined as relative motion occurring at the articular surface between the femoral component and the polyethylene bearing. Bearing axial rotation was defined as relative motion occurring at the distal backside surface between the polyethylene bearing and the tibial baseplate. Positive axial rotation corresponded to femoral internal rotation (tibial external rotation) and negative axial rotation corresponded to femoral external rotation (tibial internal rotation).

### Statistical methods

The primary endpoints of this study were knee axial rotation motion during passive knee flexion, including the magnitude of total axial rotation, articular axial rotation, and bearing axial rotation. The normal and outlier categories within each of the two groups defined by the CT measurements (Anatomic Landmarks; Rotational Mismatch) were compared for differences in clinical factors (patients’ age, weight, body mass index, Knee Society Scores), limb alignment (mechanical axis), and TKR rotational alignment. The relationships between axial rotation motion and flexion angle for the normal and outlier categories within each group also were compared. Sample size was determined in the initial clinical study [15] and was based on the ability to detect differences of 5 degrees in the components’ rotational alignment. Statistical analysis software (SigmaStat version 2.03, SPSS Inc., Chicago, Illinois USA) was used for data processing to execute analysis of variance with appropriate post-hoc multiple comparisons, including non-parametric methods when applicable.

## Results

Thirteen subjects were excluded from the kinematic assessments due to off-screen alignment during fluoroscopic imaging (2 subjects), failure to mark the principle point in the fluoroscopic images (8 subjects), and implantation of mobile polyethylene bearings without radiopaque markers (3 subjects). Rotational alignment and axial rotation motion are reported for the remaining 67 subjects (Tables 1 and 2). Significant differences in the patients’ age, weight, body mass index, Knee Society Scores and pre- and post-operative limb alignment were not detected between the nominal and outlier category subgroups in either the Anatomic Landmarks (p > 0.05) or the Rotational Mismatch (p > 0.05) groups. 

**Table 2:** Rotational alignment of components (median, range) measured from postoperative CT images for alignment groups defined with respect to anatomic landmarks (surgical transepicondylar axis, medial third of the tibial tuberosity) and rotational mismatch between femoral and tibial components

**Alignment group**		**n (% total)**	**Rotational alignment (°)**		
			**Femoral component**	**Tibial component**	**Femoral-tibial mismatch**
Anatomic Landmarks	Nominal	46/67 (69%)	0.6 (−2.8 – 2.7)	0.0 (−6.5 – 9.9)	0.4 (−9.5 – 10.6)
	Outliers	21/67 (31%)	1.2 (−3.4 – 3.8)	12.0 (−14.9 – 26.0)	−0.5 (−13.3 – 14.4)
			p = 0.554	p = 0.002	p = 0.846
Rotational Mismatch	Nominal	44/67 (66%)	0.6 (−3.4 – 3.8)	0.0 (−13.3 – 26.0)	−0.2 (−5.0 – 4.6)
	Outliers	23/67 (34%)	0.9 (−3.1 – 3.5)	0.0 (−14.9 – 20.9)	6.2 (−13.0 – 14.4)
			p = 0.659	p = 0.084	p = 0.146

Positive or negative rotational alignment corresponded to internal or external component alignment, respectively. Positive rotational mismatch corresponded to femoral internal rotation relative to the tibial component and negative rotational mismatch corresponded to femoral external rotation.

Based on CT measurements, approximately one-third of the TKR had rotational alignment outside of defined tolerances for component alignment in both the Anatomic Landmarks and Rotational Mismatch groups (Table 2). Tibial components showed a greater variance in rotational alignment compared to femoral components and contributed to a greater proportion of TKR identified as outliers. There were 16 (24%) TKR with isolated malrotation of the tibial component, 4 (6%) with isolated malrotation of the femoral component, and 1 (1%) with malalignment of both the tibial and femoral components. Ten TKR were identified as being outliers in both the Anatomic Landmark group and the Rotational Mismatch group. Outlier TKR in the Anatomic Landmarks group had 12.0° more tibial internal rotation alignment compared to nominal TKR. Outlier TKR in the Rotational Mismatch group included nine TKR with negative mismatch (femoral external rotation relative to the tibial component) and 14 TKR with positive mismatch (femoral internal rotation relative to the tibial component), resulting in 6.4° more femoral-tibial mismatch biased toward tibial external rotation (femoral internal rotation) compared to the nominal TKR.

**Figure 3:**
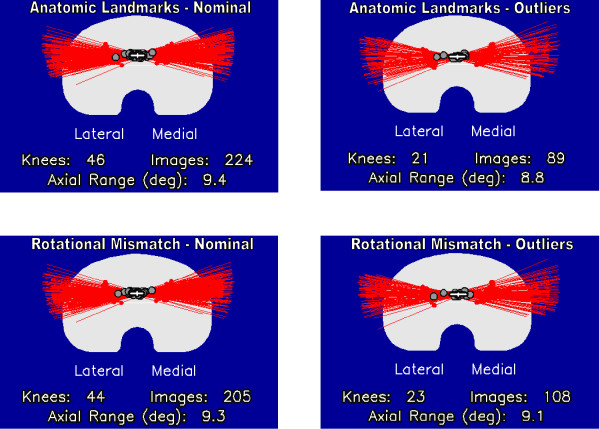
**Axial rotation motion of the polyethylene bearings about the central peg on the tibial baseplates. **The red lines indicate the rotational motion of the polyethylene mobile bearing axis projected onto the tibial baseplate for each flexion position. The gray circles indicate the computed center of rotation for every individual TKR, and the white cross indicates the mean and standard deviation of the centers of rotation. There were no significant differences between the nominal and outlier TKR in each group.

**Figure 4:**
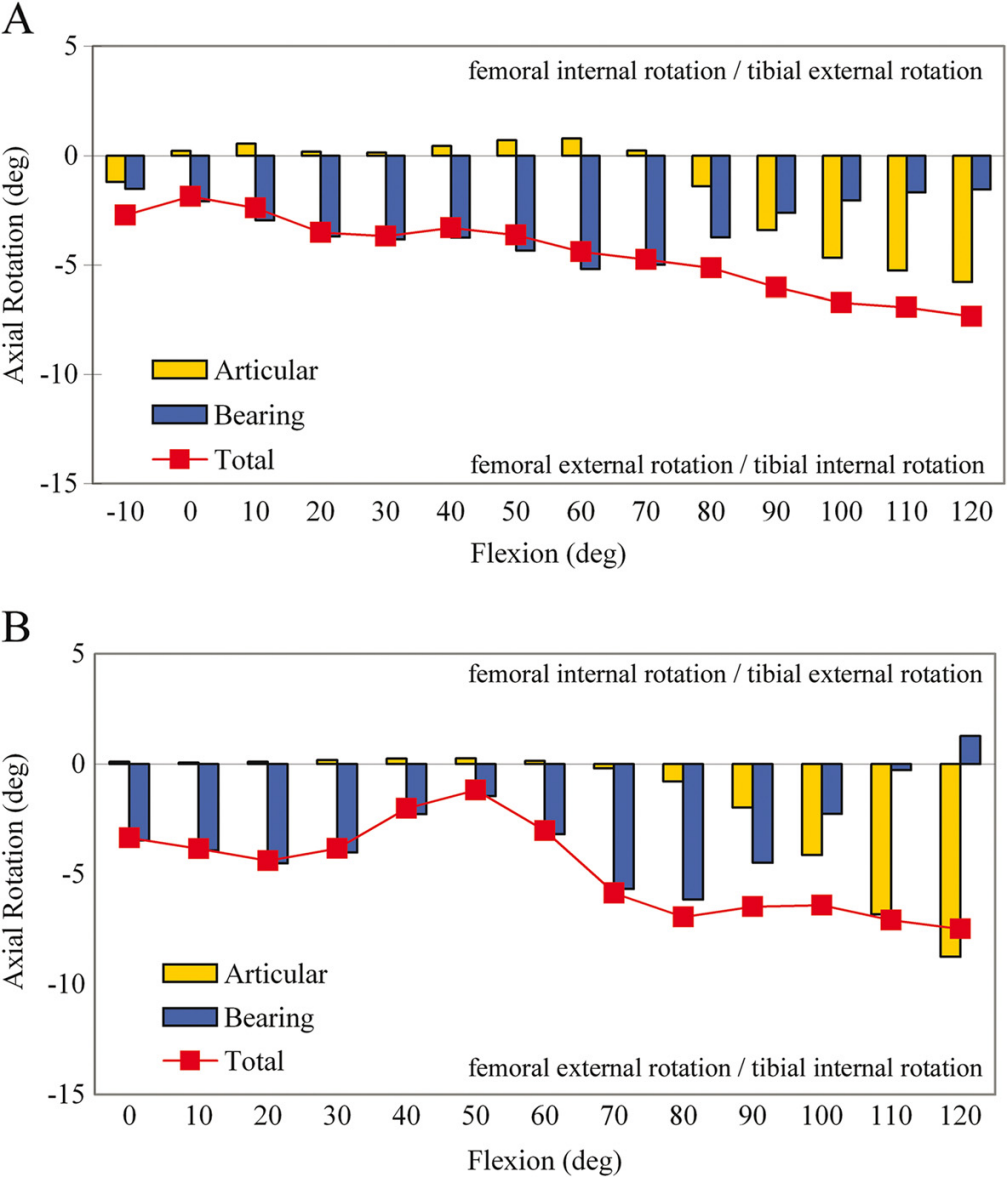
**Axial rotation kinematics in the Anatomic Landmarks group. **Axial rotation kinematics in TKR categorized as **A**) nominal and **B**) outliers in the Anatomic Landmarks group. Relative motion between the femoral component and tibial baseplate (total axial rotation), between the femoral component and polyethylene bearing (articular axial rotation) and between the polyethylene bearing and tibial baseplate (bearing axial rotation) were distinguished.

**Figure 5:**
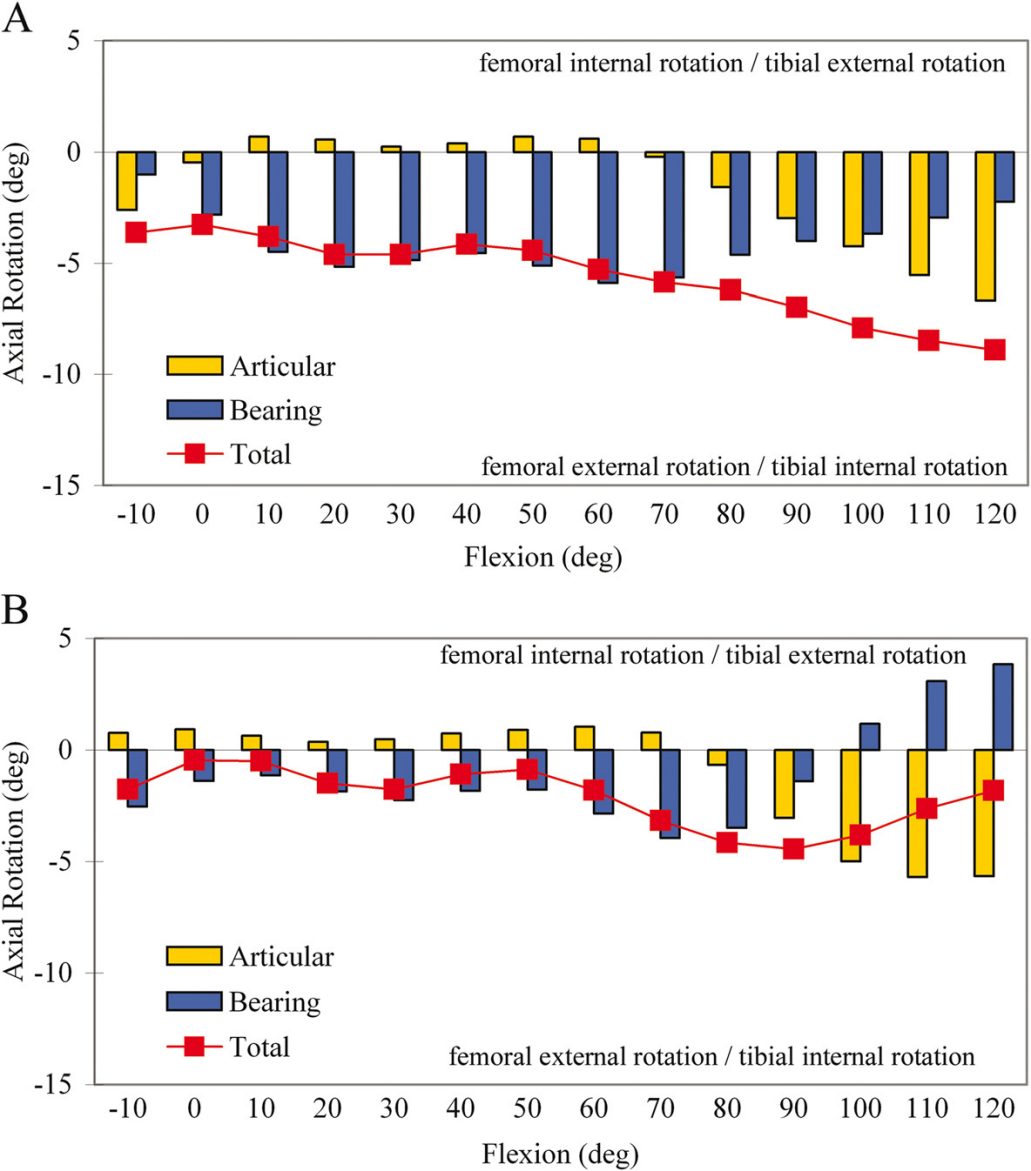
**Axial rotation kinematics in the Rotational Mismatch Group. **Axial rotation kinematics in TKR categorized as **A**) nominal and **B**) outliers in the Rotational Mismatch Group. Relative motion between the femoral component and tibial baseplate (total axial rotation), between the femoral component and polyethylene bearing (articular axial rotation) and between the polyethylene bearing and tibial baseplate (bearing axial rotation) were distinguished.

Based on measurements from fluoroscopic images, combined motion of the femoral component on the polyethylene articular surface and the bearing on the tibial insert contributed to the total axial rotation motion observed in all TKR. When averaged over the entire flexion range, there were no significant differences in the magnitude of total, articular or bearing axial rotation for TKR categorized as nominal or outlier in either the Anatomic Landmarks (p > 0.05) or Rotational Mismatch groups (p > 0.05). All groups experienced approximately 9° of axial rotation of the polyethylene bearing about the central metal peg on the tibial baseplate (Figure 3). However, different patterns of rotational motion occurred at different angles within the flexion range (Figures 4 and 5). 

In the Anatomic Landmarks group, different patterns of axial rotation motion occurred over the flexion range for the nominal and outlier groups (Figure 4). Among TKR categorized as nominal (46 knees), there was a gradual increase in total axial rotation with flexion, consistent with increasing femoral external rotation. In general, axial rotation from 0° to approximately 80° occurred primarily due to external rotation of the polyethylene bearing on the tibial baseplate. However, for TKR categorized as outliers (21 knees), there was a distinct transition from external to internal rotation from 20° to 50° of flexion and a second distinct transition into external rotation from 50° to 80° of flexion (Figure 4). Beyond 80°, both nominal and outlier TKR showed combined polyethylene bearing axial rotation and external rotation of the femoral component on the polyethylene articular surface, with the latter dominating the motion pattern. Significant differences in total axial rotation for the nominal and outlier groups were not detected (p > 0.05) for the increments of flexion. However, statistical power was limited (β<0.8) for these comparisons and the lack of observed statistical differences should be interpreted with caution.

**Figure 6:**
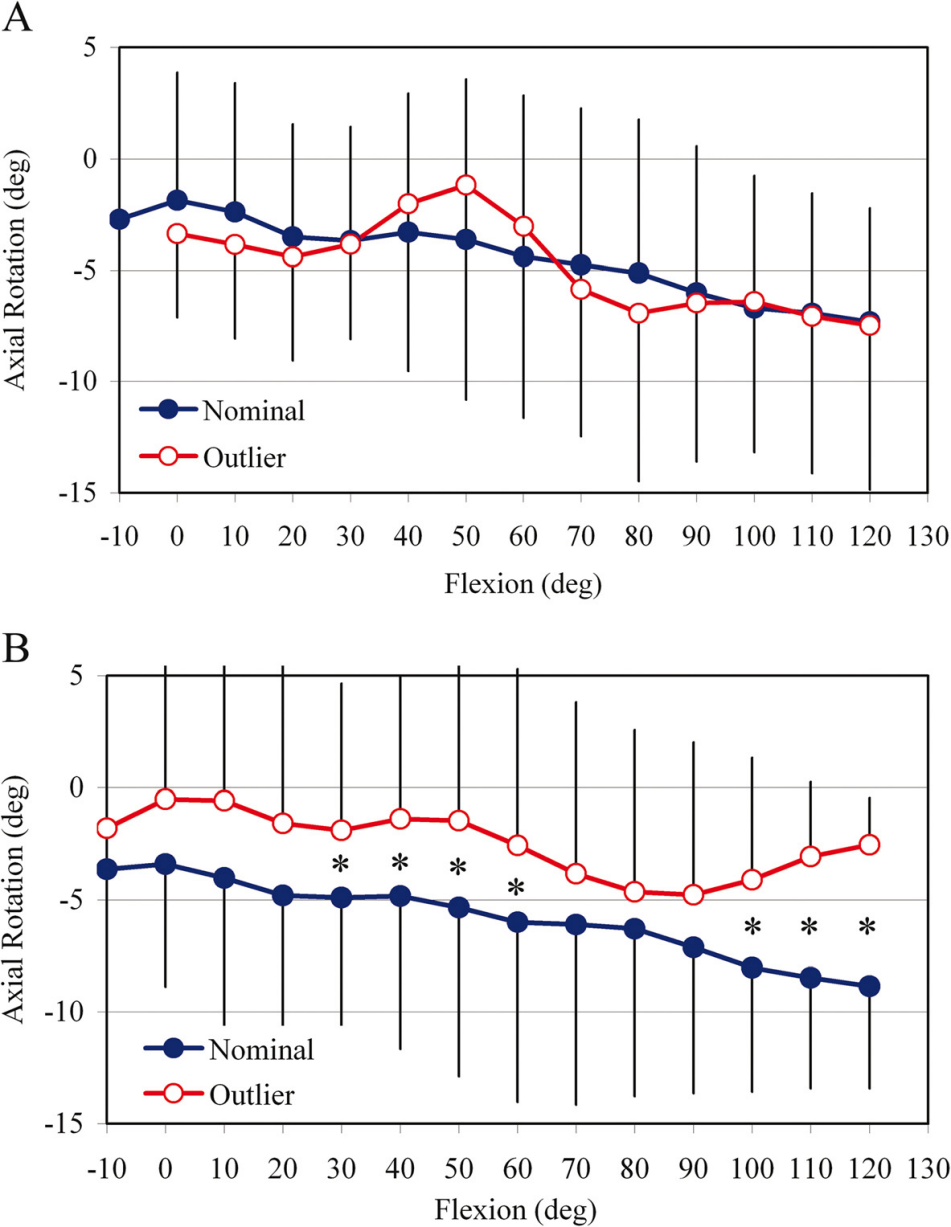
**Direct comparison of total axial rotation kinematics. **Relative motion between the femoral component and tibial baseplate (total axial rotation) for TKR in the **A**) Anatomic Landmarks Group and **B**) Rotational Mismatch Group. * = significant difference between nominal and outlier TKR in each group (ANOVA, p ≤ 0.05).

In the Rotational Mismatch group, differences between the nominal and outlier categories were largely due to opposite rotations between the femoral component and polyethylene bearing noted in the outlier category (Figure 5). Among TKR categorized as nominal (44 knees), there was a gradual increase in total axial rotation with flexion, consistent with increasing femoral external rotation. Axial rotation from 0° to 80° of flexion primarily occurred with external rotation of the polyethylene bearing on the tibial baseplate. Axial rotation from 90° to 120° of flexion occurred with combined polyethylene bearing axial rotation and external rotation of the femoral component on the polyethylene articular surface, with the latter dominating the motion pattern. For TKR categorized as outliers (23 knees), the femoral component internally rotated in early to mid flexion (−10° to 70°) and then externally rotated in later flexion (≥80°). In contrast, the polyethylene bearing showed external rotation in early to mid flexion (10° to 70°) and then internal rotation at flexion >70°. Significant differences in total axial rotation for the nominal and outlier groups occurred for increments of flexion from 30° to 60° (p = 0.05) and 100° to 120° (p < 0.001) (Figure 6). Dividing the outliers in the Rotational Mismatch group into TKR with negative mismatch and TKR with positive mismatch revealed nearly identical total axial rotation motion from 10° to 90°, with no significant differences over the flexion range (ANOVA, p > 0.05). This finding suggests that both positive and negative mismatch similarly alter femoral-tibial axial rotation relative to TKR with nominal mismatch. 

## Discussion

This study combined CT and fluoroscopic imaging during flexion to address the consequences of component malalignment on the *in vivo* motion of mobile-bearing TKR. Prosthesis rotational alignment outside of defined surgical tolerances significantly affected the knee axial rotation motion and bearing motion during passive flexion. In these subjects with mobile-bearing TKR, axial rotation was generally accomplished in two phases over the passive flexion range, dominated by polyethylene bearing rotation on the tibial component in early to mid-flexion and then femoral component rotation on the polyethylene articular surface in later flexion (Figures 4 and 5). However, TKR with rotational alignment outside of surgical norms presented different patterns of axial rotation motions, including distinct transitions in bearing rotational motion during mid-flexion, lower magnitudes of total external rotation, and opposite rotations of the femur-bearing and bearing-baseplate articulations (Figures 4 and 5).

Intraoperative assessment of passive range of motion and CT imaging of the components’ alignment proved useful for measuring *in vivo* tibial-femoral kinematics and objectively categorizing the nominal and outlier alignment groups after mobile-bearing TKR. A limitation with this study is that the measured kinematics are reflective of passive knee flexion, without muscle contraction or weight-bearing by the patient. This motion was evaluated in an effort to capture the effect of surgical technique and rotational alignment, without adding variability due to patient habitus, dynamic activity and possible pain. Furthermore, using slow and controlled movement of the knee eliminated measurement difficulties that can occur with motion blur in the image frames [25].

This mobile-bearing prosthesis design accomplished axial rotation in two phases, including external rotation of the polyethylene bearing on the tibial baseplate in early to mid-flexion and external rotation of the femoral component on the polyethylene articular surface at flexion >80°. Abrupt transitions in bearing rotation were observed and were most pronounced for outlier TKR in both the Anatomic Landmarks and Rotational Mismatch groups (Figures 4 and 5). Bearing motion did not always follow femoral motion for this activity and nonconforming mobile bearing design, consistent with other *in vivo* studies of bearing motion [25,36,39]. These data characterize a decoupling of the femoral and tibial components’ rotations, similar to the patterns observed in a dynamic musculoskeletal model of mobile-bearing TKR during simulated squatting [40].

Maintaining rotational mismatch within ±5° during TKR provided for controlled femoral external rotation motion occurring with passive flexion. In contrast, bearing motions in the mid-flexion range of motion were distinctly different among outlier TKR in both the Anatomic Landmarks and Rotational Mismatch groups compared to nominal TKR (Figures 4 and 5). This may have consequences for dynamic activities that demand stability during mid-flexion when joint loads due to muscle contraction are high. Furthermore, external rotation was essentially arrested beyond 80° in Anatomic Landmarks outliers, which can interfere with patella function [14,18,34,40]. In the Rotational Mismatch group, outlier TKR alignment was biased more than 6° toward femoral internal rotation relative to the tibial component (Table 2), resulting in significantly less total axial rotation (decreased external rotation motion) compared to nominal TKR (Figure 5). A similar reduction in axial rotation motion with femoral-tibial component mismatch biased toward femoral internal rotational alignment has been observed during *in-vitro* testing of cadaver limbs loaded to simulate rising from a chair [34].

The nominal tolerances for component alignment and rotational mismatch in the transverse plane remain under debate [8,11-16,18-22]. In the current study, TKR were categorized as nominal if component alignment relative to anatomic landmarks was within ±3° for femoral components and within ±10° for tibial components, and if relative femoral-tibial mismatch was within ±5°. These ranges were established based on reported surgical precision for achieving targeted component alignment [10,15,16] and the magnitude of deviation from optimal alignment that has been associated with clinical and biomechanical complications [18,20,31-34].

Femoral component alignment deviating from ±3° was considered as outlier alignment since it does not represent precise surgical technique and has the potential to contribute to poor outcomes. Several studies report that ±3° precision for femoral component rotation is readily achieved in more than 85% of TKR [10,15,16]. Femoral component rotation exceeding approximately ±5° has been associated with clinical complications, [33] including pain [32] and patellar failure. [18] It is recognized that precise tibial component axial rotation relative to anatomic landmarks is difficult to achieve. Tibial component alignment deviating from ±10° was considered as outlier alignment since it exceeds surgical norms and has the potential to contribute to poor outcomes. Reported alignment precision for tibial components exceeds ±3° in approximately 50% of TKR [10] and exceeds ±10° in approximately 30% of TKR [15]. Absolute mean deviations of 3° to 8° of tibial component axial rotation alignment have been reported, [10,15] with pain [32] and patellar dislocation and failure [18] associated with deviations exceeding approximately 10°.

While several studies report combined rotation and rotational mismatch between the femoral and tibial components after TKR [14,18,32,41], few report clinical consequences associated with these parameters. Adverse consequences associated with approximately 10° of combined rotation or rotational mismatch include no improvement in Knee Society function scores, [41], knee pain, [32] and patellar dislocation or failure [18]. In the current study, 7 of the 23 TKR categorized as outliers in the Rotational Mismatch group had rotational mismatch exceeding ±10° and those patients previously were reported to exhibit no functional improvement [41]. Expanding the current analysis to include TKR with rotational mismatch exceeding ±5° shows that even smaller magnitudes of mismatch can have significant biomechanical consequences (Figure 6B).

Obtaining alignment within the above defined nominal ranges provided for controlled knee axial rotation (Figure 4 and 5). However, these tolerances were exceeded in 31% and 34% of the TKR when evaluated relative to anatomic landmarks and rotational mismatch, respectively (Table 2). Rotational alignment of the tibial components proved especially variable and contributed to these relatively high percentages of outliers, similar to our previous report, [15] as surgical techniques referencing the tibial tubercle have proven inconsistent [15-17]. The observed variations in surgical rotational alignment provide some explanation for the highly variable bearing motions that have been observed *in vivo* for various mobile-bearing TKR designs [24-27,36-39].

Clinical consequences for TKR patients with isolated and combined internal rotation alignment of the femoral and tibial components include anterior knee pain and patellar complications [14,18,21,32]. In a series of failed TKR with patellofemoral complications, 3°–8° of internal rotation malalignment was correlated with patellar subluxation and 7°–17° of internal rotation malalignment was correlated with early patellar dislocation or late prosthesis failure [18]. Barrack, et al. [14] found 6.8° more internal rotational alignment of the tibial component in patients with anterior knee pain, both with and without patellar resurfacing, compared to control patients without pain. Compared to nominal TKR in the current study, outliers in the Anatomic Landmarks group showed a bias of 12° of internal rotation of the tibial component, and outliers in the Rotation Mismatch group showed a bias of 6.4° more femoral internal rotation (tibial external rotation) malalignment (Table 2). Careful monitoring of the mid-term clinical outcomes for all patients in the current study cohort is ongoing, with preliminary data showing a trend toward worse Knee Society function scores in patients with more than 10° of relative rotational alignment between the femoral and tibial components at a median follow-up time of 20 months [41]. Therefore, contrary to some studies suggesting that mobile-bearing TKR designs compensate for errors in rotational alignment [17,19,21-24], patients with mobile-bearing TKR can experience measurable kinematic differences and worse functional outcomes [41] when rotational alignment is outside of defined surgical norms.

## Conclusions

Axial rotation motion was generally accomplished in two phases, dominated by polyethylene bearing rotation on the tibial component in early to mid-flexion and then femoral component rotation on the polyethylene articular surface in later flexion. Maintaining relative rotational mismatch within ±5° during TKR provided for controlled knee axial rotation during flexion. TKR with rotational alignment outside of defined surgical norms experienced measurable kinematic differences and presented different patterns of axial rotation motions during passive knee flexion. These findings support previous studies [14,18,21,32,41] linking prosthesis rotational alignment with inferior clinical and functional outcomes.

## Competing interests

The authors declare that they have no competing interests.

## Authors’ contributions

MH participated in the study design, completed analysis of the fluoroscopic images, performed statistical analysis, and helped to draft the manuscript. SB developed the shape matching technique for measuring knee kinematics, provided technical support, and helped to draft the manuscript. SK participated in the study design and coordination, completed TKR surgery, acquired CT and fluoroscopic images, provided clinical assessments, and helped to draft the manuscript. JL conceived of the study, completed TKR surgery, acquired CT and fluoroscopic images, provided clinical assessments, measured alignment from CT images, and helped to draft the manuscript. All authors have read and approved the final manuscript.

## Pre-publication history

The pre-publication history for this paper can be accessed here:

http://www.biomedcentral.com/1471-2474/13/206/prepub
